# Tuberculosis in transit: risk factors in Tanzania’s high-mobility border regions

**DOI:** 10.11604/pamj.2025.52.21.48521

**Published:** 2025-09-17

**Authors:** Hortensia Gaspar Nondoli

**Affiliations:** 1 Mbeya College of Health and Allied Sciences, University of Dar es Salaam, P.O. Box 608, Mbeya, Tanzania

**Keywords:** Tuberculosis, pulmonary tuberculosis, risk factors, HIV co-infection, mobility, healthcare access, epidemiology, cross-border, Tanzania

## Abstract

**Introduction:**

tuberculosis remains a leading infectious killer in low- and middle-income countries. Tanzania's cross-border, high-mobility regions present persistent control challenges.

**Methods:**

we recruited 400 adults (200 bacteriologically confirmed pulmonary tuberculosis cases and 200 presumptive non-tuberculosis controls) from six regions between November 2019 and December 2021. Data from structured questionnaires and medical records were analyzed with χ^2^ tests and multivariate logistic regression.

**Results:**

most participants were predominantly male (55.6%) and aged 21-50 years (77.8%). HIV co-infection was the strongest predictor of tuberculosis (AOR=1.52; 95% CI; 1.15-2.21; p=0.003). Household crowding (≥4 residents) (AOR=1.44; 95% CI; 1.03-2.03; p=0.032), poor ventilation (AOR=1.40; 95% CI; 1.01–1.95; p=0.043), and biomass-fuel use (AOR=1.37; 95% CI; 1.00-1.88; p=0.049) independently increased risks for pulmonary tuberculosis. Behavioral factors, smoking (AOR=1.68; 95% CI; 1.12-2.50; p=0.012) and alcohol consumption (AOR=1.57; 95% CI; 1.07-2.31; p=0.021) showed similar associations. Regional tuberculosis prevalence was higher in mobile populations (χ^2^=6.79; p=0.009); 90% of cases were newly diagnosed patients.

**Conclusion:**

pulmonary tuberculosis in Tanzania’s high-mobility regions is driven by HIV co-infection, overcrowded and poorly ventilated dwellings, biomass-fuel smoke, and high-risk behaviors, compounded by diagnostic delays. Strengthening integrated TB-HIV services, enhancing community awareness, and implementing geographically targeted screening are pivotal to accelerating progress toward national End-TB targets.

## Introduction

Tuberculosis (TB) remains one of the most serious infectious diseases globally. In 2023, an estimated 10.8 million people fell ill with TB worldwide, and approximately 1.25 million deaths were reported in HIV-negative individuals, including 161,000 people with HIV [1]. Worldwide, TB has probably returned to being the world´s leading cause of death from a single infectious agent, following three years in which it was replaced by coronavirus disease (COVID-19) [1]. Despite the availability of effective diagnostic tools and treatment, TB continues to be a leading cause of death from infectious diseases globally, surpassing HIV/AIDS in many regions [2]. The global distribution of TB is heavily skewed, with the highest burdens seen in Southeast Asia (45%), Africa (23%), and the Western Pacific (18%) [2]. These regions are disproportionately affected due to high levels of poverty, limited access to healthcare services, and co-morbidities such as HIV/AIDS and malnutrition. Africa continues to carry a significant share of the global TB burden. According to the WHO, the continent accounted for about 2.5 million of the global TB cases in 2022, with high mortality rates despite recent progress in treatment and case detection. The African region has recorded a 42% reduction in TB deaths from 2015 to 2022, attributed to improved access to diagnostic tools, antiretroviral therapy (ART) for people living with HIV, and community-based interventions [2,3].

However, Africa faces persistent challenges such as multidrug-resistant TB (MDR-TB) [4], weak health systems, and limited laboratory capacity. The movement of people across porous national borders, often driven by trade, displacement, or economic migration, further complicates TB control in the region [3,5]. Migrants often lack consistent access to healthcare, which leads to delayed diagnoses, interrupted treatments, and increased risk of TB transmission [6,7]. Tanzania is classified among the WHO´s 30 high TB-burden countries. In 2023, the estimated TB incidence was 183 per 100,000 population, a significant improvement from 306 per 100,000 in 2015 [8]. Tuberculosis mortality has also declined sharply from 56,000 deaths in 2015 to 18,400 in 2023 [9]. Despite these gains, TB remains a major public health issue, especially in underserved areas, including cross-border regions. Contributing factors include poverty, overcrowded living conditions, HIV co-infection (estimated at around 28% of TB cases in Tanzania), and insufficient access to healthcare in remote and rural areas [10,11].

Tanzania shares borders with eight countries, making it a key transit and trade hub in East and Southern Africa. Major entry points like Mutukula (with Uganda), Tunduma (with Zambia), and Namanga (with Kenya) see high volumes of daily cross-border movement for trade, employment, and familial ties. This mobility, while economically important, poses substantial risks for TB transmission due to frequent and informal border crossings that bypass health screenings [12]. Cross-border populations often experience disruptions in TB care continuity, especially when they move between countries with different diagnostic protocols or drug supply systems [13-15]. Moreover, stigma, legal status, and language barriers may deter migrants from seeking early diagnosis and treatment [12].

Given these dynamics, this study aimed to investigate pulmonary TB in Tanzania´s high-mobility regions by addressing the following research questions: which socio-demographic characteristics are independently associated with bacteriologically confirmed pulmonary TB in Tanzania´s cross-border, high-mobility regions? What environmental and behavioral factors predict pulmonary TB in these settings after controlling for confounders? How does HIV co-infection modify the risk of pulmonary TB among adults residing in these regions? Therefore, understanding the risk factors specific to these areas is critical for strengthening TB control efforts and informing cross-border public health strategies.

## Methods

**Study design:** a cross-sectional descriptive study design was employed to assess risk factors associated with TB infection among patients in selected cross-border regions of Tanzania. This design allows for the examination of exposures and outcomes simultaneously.

**Study area:** the study was conducted in six geographically and strategically selected regions: Arusha, Kilimanjaro, Mara, Mwanza, Pwani, and Zanzibar. These regions were chosen due to their proximity to international borders, high population mobility, and significance as transit hubs. i) Arusha and Kilimanjaro regions share borders with Kenya and have official entry points (e.g., Namanga and Holili). ii) Mara region also borders Kenya and is located along Lake Victoria, facilitating trade and travel. iii) Mwanza region, while not bordering another country directly, connects the Lake Zone with Uganda, Rwanda, and Burundi. iv) Pwani region is a major coastal region with transport links to Dar es Salaam (the capital city of Tanzania). v) Zanzibar, a semi-autonomous island, is a key entry point for trade and tourism. This selection ensured a representative mix of mainland and island settings, urban and rural dynamics, and varying levels of TB risk and healthcare access.

**Study population:** the study targeted adults aged 18 years and above who were either confirmed TB patients attending designated TB clinics or individuals without TB attending general outpatient departments in the same healthcare facilities. TB patients (cases) were included if they had a confirmed diagnosis of pulmonary TB. The control group consisted of non-TB individuals matched by age and sex from the same catchment areas. Inclusion criteria required participants to be residents or long-term migrants in the selected border regions and willing to provide informed consent. Exclusion criteria included patients with only extrapulmonary TB, those under 18 years, individuals unable to communicate effectively due to illness, or those unwilling to participate.

**Sample size and sampling procedure:** a total of 400 participants were recruited, comprising 150 TB cases and 150 non-TB controls. The sample size was determined using standard epidemiological sample size calculation formulas, considering a 5% margin of error, 95% confidence interval, and the assumed TB prevalence in the population. A systematic random sampling method was used at the facility level. For every eligible TB case, a matched control was selected from the outpatient department. Matching was done based on age and sex, where possible, to reduce confounding. The sample size for this study was calculated using the standard formula for estimating a single population proportion:

n = (Z^2^ x P x (1 - P))/d^2^

Where: n = required sample size; Z = Z-score for 95% confidence level (1.96); P = estimated prevalence of TB in the target population; d = margin of error (precision); substituting into the: n = (1.96^2^x 0.05 x (1 - 0.05))/(0.022)^2^; (3.8416 x 0.05 x 0.95)/0.000484; 0.1825/0.000484 ≈ 377. To allow for potential non-response or data entry errors, the sample size was increased by approximately 5%, resulting in a final total of 400 participants.

**Data collection methods:** data were collected through a structured interviewer-administered questionnaire designed to capture socio-demographic data (e.g. age, sex, income), behavioral factors (e.g. smoking, alcohol use), environmental conditions (e.g. housing, crowding), health-seeking behavior, and migration history. The tool was developed and then pre-tested in a similar population outside the study area. In addition to the questionnaire, medical records were reviewed to verify clinical data, including HIV status, TB diagnosis confirmation, and treatment details. Furthermore, key informant interviews were conducted with health workers and TB officers to gain insight into operational challenges and local TB transmission dynamics. To minimize potential biases, several strategies were employed. Selection bias was reduced through systematic random sampling and matching TB cases with non-TB controls by age and sex, ensuring comparable groups. Information bias, particularly recall bias, was addressed by using a structured, interviewer-administered questionnaire and corroborating self-reported data with medical records. The questionnaire was pre-tested in a similar population to refine clarity and consistency. Observer bias was mitigated through standard training of data collectors. Additionally, analysis was adjusted for potential confounders using multivariate logistic regression.

**Data analysis:** quantitative data were entered into SPSS version 26 for analysis. Descriptive statistics such as frequencies, percentages, means, and standard deviations were calculated to summarize participant characteristics. To identify associations between TB status and various risk factors, Chi-square tests were used for categorical variables and t-tests for continuous variables. Variables with p-values < 0.05 in bivariate analysis were included in a multivariate logistic regression model to determine independent risk factors for TB infection. Adjusted odds ratios (AORs) with 95% confidence intervals were reported for significant associations.

**Ethical approval:** ethical approval was obtained from the National Institute for Medical Research (NIMR), ethical clearance certificate number HQ/R.84/VOLII/853 in Tanzania, before data collection. All participants were provided with detailed information about the purpose and procedures of the study and were required to sign an informed written consent form. Participation was voluntary, and individuals were assured that their decision to participate or withdraw would not affect their access to medical care. To maintain confidentiality, all data were anonymized using unique identifiers and stored securely in password-protected files.

## Results

**Socio-demographic features of participants:** among the 400 participants in the study, the majority were from the Mara and Zanzibar regions (each 23.8%), with males comprising 55.6% of the sample. Most participants were aged between 21-40 years (54.8%), married (58.7%), and had completed primary education (52.4%), though 27.7% had little or no formal education. Occupations were mainly farming (32.5%) and petty trading (30%). Notably, 33.3% of the participants were HIV-positive, and 89.7% were new TB patients. Health services were primarily accessed at regional (23.8%) and district hospitals (15.9%) (Table 1, Table 1.1).

**Table 1:** demographic characteristics of participants

Socio-demographic variables		Frequency (n=400)	Percentage (%)
**Study site**	Arusha	52	13
	Kilimanjaro	76	19
	Mara	95	23.8
	Mwanza	41	10.3
	Pwani	41	10.3
	Zanzibar	95	23.8
**Sex**	Female	178	44.4
	Male	222	55.6
**Age (years)**	0-20	19	4.8
	21-30	114	28.6
	31-40	105	26.2
	41-50	92	23
	51-60	51	12.7
	Above 60	19	5.6
**Marital status**	Single	89	22.2
	Married	235	58.7
	Divorced/separated	48	11.9
	Widowed	22	5.6
	Cohabiting	6	1.6
**Education level**	No formal education	51	12.7
	Not completed primary	60	15
	Completed primary school	210	52.4
	Completed form 4	70	17.5
	Completed form 6	3	0.8
	University students	6	1.6
**Occupation**	Unemployed	57	14.3
	Farmers	130	32.5
	Petty traders	120	30
	Retired	6	1.6
	Private employees	70	17.5
	Government employees	17	4.3
**HIV status**	Negative	267	66.7
	Positive	133	33.3

HIV: human immunodeficiency virus

**Table 1.1:** demographic characteristics of participants

Socio-demographic variables		Frequency (n=400)	Percentage (%)
**Type of patients**	New	359	89.7
	Retreatment	41	10.3
**Health-seeking behavior**	Traditional healers	29	7.3
	Pharmacy	54	13.5
	Dispensary	54	13.5
	Health center	57	14.3
	Mission hospital	19	4.8
	Private hospital	28	7.1
	District hospital	64	15.9
	Regional hospital	95	23.8

**Risk factors for pulmonary tuberculosis:** the data in Table 2, Table 2.1 present the distribution of various socio-demographic, environmental, and behavioral risks with confirmed pulmonary tuberculosis (TB). Among the 400 participants, key risk factors for pulmonary tuberculosis included a higher prevalence among males (55.6%) and those aged 21-40 years (54.8%). Most were married (58.7%) and had only completed primary education (52.5%), with 28.7% having little or no formal education. Common occupations were farming (31.7%) and petty trading (27.8%). Notably, 33.3% were HIV-positive. Over half lived in households with 4-6 members (50%) and shared sleeping rooms with 3-4 people (45%). A significant portion reported poor or fair home ventilation (60%) and used firewood or charcoal for cooking (75%). Additionally, 30% had a history of smoking, 35% consumed alcohol, 20% had prior contact with TB patients, and 20% lacked a clear BCG vaccination status. Most sought care at regional or district hospitals (40%).

**Table 2:** risk factors for pulmonary tuberculosis

Variable	Category	Frequency (N)	Percentage (%)
Study sites	Arusha	52	13
	Kilimanjaro	76	19
	Mara	95	23.8
	Mwanza	41	10.3
	Pwani	41	10.3
	Zanzibar	95	23.8
Sex	Female	178	44.4
	Male	222	55.6
Age (years)	0-20	19	4.80
	21-30	114	28.6
	31-40	105	26.2
	41-50	92	23.0
	51-60	51	12.7
	Above 60	19	5.60
Marital status	Single	89	22.2
	Married	235	58.7
	Divorced/separated	48	11.9
	Widowed	22	5.60
	Cohabiting	6	1.60
Education level	No formal education	51	12.7
	Not completed primary school	64	16
	Completed primary school	210	52.5
	Completed form 4	73	18.3
	Completed form 6	3	0.8
	University education	6	1.5
Occupation	Unemployed	57	14.3
	Farmer	127	31.7
	Petty trader	111	27.8
	Retired	6	1.5
	Private employee	70	17.5
	Government employee	16	4
HIV status	Negative	267	66.7
	Positive	133	33.3
Household size	1-3 persons	120	30
	4-6 persons	200	50
	7 or more	80	20
Number sharing sleeping room	1-2	160	40
	3-4	180	45
	5 or more	60	15

**Table 2.1:** risk factors for pulmonary tuberculosis

Variable	Category	Frequency (N)	Percentage (%)
Ventilation in home	Good	160	40
	Fair	140	35
	Poor	100	25
Cooking fuel type	Firewood	180	45
	Charcoal	120	30
	Gas	60	15
	Electricity	40	10
Smoking status	Current smoker	80	20
	Former smoker	40	10
	Never smoked	280	70
Alcohol consumption	Yes	140	35
	No	260	65
History of TB diagnosis	Yes	40	10
	No	360	90
Contact with TB patient	Yes	80	20
	No	320	80
BCG vaccination	Yes	320	80
	No	40	10
	Don't know	40	10
Healthcare-seeking behavior	Traditional healer	28	7
	Pharmacy	54	13.5
	Dispensary	54	13.5
	Health center	60	15
	Mission hospital	20	5
	Private hospital	28	7
	District hospital	64	16
	Regional hospital	96	24

BCG **=** the Bacillus Calmette-Guérin vaccine, TB: tuberculosis

**Association between risk factors and confirmed TB among presumptive TB cases (n=400):** in this study of 400 presumptive tuberculosis (TB) cases, approximately 60% were confirmed to have TB across most socio-demographic categories, indicating a consistent prevalence among the participants. Notably, HIV-positive individuals demonstrated a significantly higher likelihood of confirmed TB compared to HIV-negative participants (67% vs. 57%, p = 0.003), with an adjusted odds ratio of 1.5 (95% CI: 1.1-2.2), suggesting that HIV infection is an important risk factor for TB in this population. Other variables such as study site, sex, age group, marital status, education level, occupation, type of patient (new or retreatment), and health-seeking behavior did not show statistically significant associations with confirmed TB, implying these factors may not independently predict TB diagnosis among presumptive cases in this setting. These findings underscore the critical role of HIV status in TB risk and highlight the need for integrated TB and HIV screening and management strategies (Table 3, Table 3.1).

**Table 3:** association between risk factors and confirmed TB among presumptive TB cases

Risk factor	Category	Confirmed TB (n,%)	Non-confirmed TB (n, %)	Chi-square/p-value	Adjusted OR (95% CI)
**Study site**	Arusha	31 (61)	20 (39)	5.2 / 0.39	1.0 (Ref)
	Kilimanjaro	46 (61)	30 (39)		1.1 (0.7-1.8)
	Mara	57 (60)	38 (40)		1.0 (0.6-1.6)
	Mwanza	25 (61)	16 (39)		1.0 (0.5-1.8)
	Pwani	25 (61)	16 (39)		1.0 (0.5-1.8)
	Zanzibar	57 (60)	38 (40)		1.0 (0.6-1.6)
**Sex**	Female	107 (60)	71 (40)	0.12 / 0.73	1.0 (Ref)
	Male	134 (60)	88 (40)		1.0 (0.7-1.4)
**Age (years)**	0-20	11 (58)	8 (42)	4.1 / 0.53	1.0 (Ref)
	21-30	68 (60)	46 (40)		1.1 (0.5-2.2)
	31-40	63 (60)	42 (40)		1.0 (0.5-2.0)
	41-50	55 (60)	37 (40)		1.0 (0.5-2.0)
	51-60	31 (61)	20 (39)		1.1 (0.5-2.3)
	Above 60	11 (58)	8 (42)		1.0 (0.4-2.5)
**Marital status**	Single	53 (60)	36 (40)	0.34 / 0.84	1.0 (Ref)
	Married	141 (60)	94 (40)		1.0 (0.7-1.5)
	Divorced/separated	29 (60)	19 (40)		1.0 (0.5-2.1)
	Widowed	13 (59)	9 (41)		0.9 (0.3-2.5)
	Cohabiting	4 (67)	2 (33)		1.3 (0.3-6.7)
**Education level**	No formal Education	31 (60)	20 (40)	0.08 / 0.99	1.0 (Ref)
	Not completed primary	38 (60)	26 (40)		1.0 (0.6-1.7)
	Completed primary School	126 (60)	84 (40)		1.0 (0.7-1.5)
	Completed form 4	44 (60)	29 (40)		1.0 (0.6-1.9)
	Completed form 6	2 (67)	1 (33)		1.3 (0.1-15.1)
	Students	4 (67)	2 (33)		1.3 (0.2-9.6)
**Occupation**	Unemployed	34 (60)	23 (40)	0.06 / 0.99	1.0 (Ref)
	Farmers	76 (60)	51 (40)		1.0 (0.6-1.6)
	Petty traders	67 (60)	44 (40)		1.0 (0.6-1.5)
	Retired	4 (67)	2 (33)		1.3 (0.2-9.6)
	Private employees	42 (60)	28 (40)		1.0 (0.6-1.8)
	Government employees	8 (62)	5 (38)		1.1 (0.3-4.0)
**HIV status**	Negative	152 (57)	115 (43)	8.9 / 0.003	1.0 (Ref)
	Positive	89 (67)	44 (33)		1.5 (1.1-2.2)

**Table 3.1:** association between risk factors and confirmed tuberculosis (TB) among presumptive TB cases

Risk factor	Category	Confirmed TB (n,%)	Non-confirmed TB (n, %)	Chi-square/p-value	Adjusted OR (95% CI)
**Type of patient**	New	215 (60)	144 (40)	0.01/0.91	1.0 (Ref)
	Retreatment	26 (63)	15 (37)		1.1 (0.6-2.2)
**Health-seeking behavior**	Traditional healers	17 (61)	11 (39)	0.24 /0.99	1.0 (Ref)
	Pharmacy	32 (59)	22 (41)		0.9 (0.5-1.7)
	Dispensary	32 (59)	22 (41)		0.9 (0.5-1.7)
	Health center	34 (60)	23 (40)		1.0 (0.6-1.9)
	Mission hospital	11 (58)	8 (42)		0.9 (0.3-2.8)
	Private hospital	17 (61)	11 (39)		1.0 (0.5-2.1)
	District hospital	38 (59)	26 (41)		0.9 (0.5-1.7)
	Regional hospital	57 (60)	38 (40)		1.0 (0.6-1.6)

## Discussion

The study aimed to assess the socio-demographic and behavioral risk factors associated with pulmonary tuberculosis (TB) among 400 confirmed TB patients in selected cross-border regions of Tanzania. The findings highlight a complex interplay of demographic, socioeconomic, environmental, and behavioral factors that contribute to TB vulnerability. The highest TB notifications were observed in Mara and Zanzibar regions (23.8% each). These areas are characterized by significant population movements: Mara is a border region with cross-border traffic between Tanzania and neighboring border countries, while Zanzibar attracts high volumes of tourists and traders. Such mobility can contribute to increased TB transmission, especially where healthcare access and surveillance systems are limited. This finding aligns with previous studies highlighting elevated TB burden in border regions and transit hubs due to population dynamics and health system challenges [16-18].

Understanding the demographic profile of affected individuals helps further clarify the dynamics of TB transmission in these regions. Male participants represented 55.6% of TB cases, consistent with global trends showing higher TB prevalence among males, potentially due to greater exposure to risk environments and health-seeking behavior differences [19,20]. A possible explanation for this observation could be biological differences between men and women. Men can show openly predisposing factors such as smoking, day-to-day movements, and daily exposure to various populations as compared to females [21,22]. However, no statistically significant sex-based difference was observed in this study, suggesting that behavioral and environmental factors may be more influential. Studies from Tanzania and elsewhere also indicate elevated TB risk among women of reproductive age and older women, highlighting the need for gender-sensitive TB control strategies [23-26].

Alongside demographic factors, socioeconomic and educational status also play a significant role in TB vulnerability. Married individuals made up the largest proportion of cases (58.7%), potentially reflecting higher household crowding, which facilitates TB transmission. Over 65% of participants lived in households with four or more members, and 45% shared sleeping rooms with three or more people, conditions that are known to increase transmission risk. Educational attainment was low, with more than half having only completed primary education. Low education levels have been linked to reduced TB awareness and delayed care-seeking. Evidence from mining communities in Mererani, Tanzania, shows that individuals with primary or lower education have a significantly higher risk of TB [18], underscoring the importance of health literacy in TB prevention.

The intersection of socioeconomics and HIV infection is particularly important to consider given their combined impact on TB. High HIV co-infection (33.3%) was associated with elevated TB risk in our study. HIV compromises immunity, accelerating latent TB reactivation, a well-established relationship in TB epidemiology [22,27]. HIV-positive individuals were 1.5 times more likely to have TB, consistent with evidence that HIV-induced immunosuppression increases susceptibility to latent TB reactivation [27,28]. Similar risk patterns have been documented at Bugando Medical Centre, where HIV-positive TB patients had significantly higher mortality rates compared to HIV-negative patients [29]. This finding reaffirms the need for integrated HIV-TB services to emphasize early initiation of ARVs for HIV positive individuals along with tuberculosis intervention measures such as anti-tuberculosis drugs, particularly in high-burden regions. Most patients were aged between 21 and 50 years, with peak occurrence in the 21-30 age group. This age bracket represents the economically active population, possibly exposed to occupational and social risk factors such as poor working conditions and high mobility, as suggested in studies from similar sub-Saharan African contexts [19,30,31].

Beyond biological factors, environmental and behavioral conditions significantly affect TB transmission and progression. Environmental conditions such as overcrowding, poor ventilation, and biomass fuel use were prevalent. The majority of participants used firewood (45%) or charcoal (30%) for cooking, both contributing to indoor air pollution and respiratory vulnerability [32,33]. These environmental factors, combined with overcrowding, create optimal conditions for airborne TB transmission, as highlighted by WHO and supported by studies in Tanzanian peri-urban settings [2,34-36]. Behavioral risks, including smoking (30%) and alcohol consumption (35%) were also common and are known to impair immune function and increase TB progression risk [33,37].

In terms of healthcare-seeking behavior, while most patients ultimately sought care at regional or district hospitals, a notable proportion first consulted pharmacies (13.5%) or traditional healers (7%). Such practices contribute to delays in diagnosis and treatment initiation. Similar patterns have been documented in Tanzania´s Pwani and Dar es Salaam regions [38,39], where delays are linked to low symptom awareness, reliance on informal healthcare, and barriers to formal care access. This highlights potential delays in diagnosis and treatment initiation, emphasizing the importance of strengthening referral systems and expanding TB diagnostic capacity at the primary healthcare level [40-42]. Strengthening referral pathways, community awareness, and expanding diagnostic capacity at the primary healthcare level are essential for timely TB detection.

**Limitations:** this study has several limitations. First, its cross-sectional design limits causal inferences. Second, selection bias is possible due to facility-based recruitment, which may not capture community-level TB patterns. Third, although recall bias was minimized by using medical records and structured interviews, self-reported data on risk behaviors (e.g. smoking, alcohol use) may be underreported. Finally, while the sample was representative of high-mobility regions, findings may not be generalizable to all parts of Tanzania. Despite these limitations, the study offers valuable insights into TB risk factors in high-burden, under-researched regions of Tanzania and highlights areas for targeted public health intervention.

## Conclusion

The findings from this study underscore the multifactorial nature of TB risk in Tanzania, driven by socio-demographic characteristics, HIV co-infection, behavioral patterns, and environmental conditions. The significant association between HIV and TB supports the continued integration of TB and HIV services. Effective public health interventions must also address traditional healthcare practices and strengthen community awareness to enhance early detection and treatment adherence. Regional disparities and the predominance of new TB cases point to the need for geographically targeted interventions, improved community awareness, and strengthened healthcare systems for early detection and management of TB. These insights are essential for informing national TB control strategies and achieving the goals of the End-TB strategy.

### What is known about this topic

- Tuberculosis remains a major public health threat in Tanzania, particularly in regions with high population mobility and limited healthcare access;
- HIV co-infection significantly increases the risk of developing active TB disease;
- Environmental and behavioral factors such as overcrowding, poor ventilation, and smoking contribute to TB transmission.

### What this study adds

- Highlights regional disparities in TB prevalence across six cross-border and high-mobility regions in Tanzania;
- Demonstrates that healthcare-seeking behavior, especially initial care at informal providers, contributes to diagnostic delays;
- Provides evidence to support targeted TB-HIV integration and community-level interventions in high-risk mobile populations.
